# Novel Preclinical and Radiopharmaceutical Aspects of [^68^Ga]Ga-PSMA-HBED-CC: A New PET Tracer for Imaging of Prostate Cancer

**DOI:** 10.3390/ph7070779

**Published:** 2014-06-30

**Authors:** Matthias Eder, Oliver Neels, Miriam Müller, Ulrike Bauder-Wüst, Yvonne Remde, Martin Schäfer, Ute Hennrich, Michael Eisenhut, Ali Afshar-Oromieh, Uwe Haberkorn, Klaus Kopka

**Affiliations:** 1 German Cancer Research Center (dkfz), Division of Radiopharmaceutical Chemistry, Im Neuenheimer Feld 280, Heidelberg 69120, Germany; E-Mails: o.neels@dkfz.de (O.N.); miriam.mueller@wak-gmbh.de (M.M.); u.bauder-wuest@dkfz.de (U.B.-W.); y.remde@dkfz.de (Y.R.); martin.schaefer@dkfz.de (M.S.); u.hennrich@dkfz.de (U.He.); m.eisenhut@dkfz.de (M.E.); k.kopka@dkfz.de (K.K.); 2 German Cancer Consortium (DKTK), Im Neuenheimer Feld 280, Heidelberg 69120, Germany; 3 Department of Nuclear Medicine, University of Heidelberg, Im Neuenheimer Feld 400, Heidelberg 69120, Germany; E-Mails: Ali.Afshar@med.uni-heidelberg.de (A.A.-O.); Uwe.Haberkorn@med.uni-heidelberg.de (U.Ha.)

**Keywords:** ^68^Ga-PET imaging, PSMA, HBED-CC, prostate cancer, radiopharmaceutical production, good manufacturing practice, GMP

## Abstract

The detection of prostate cancer lesions by PET imaging of the prostate-specific membrane antigen (PSMA) has gained highest clinical impact during the last years. ^68^Ga-labelled Glu-urea-Lys(Ahx)-HBED-CC ([^68^Ga]Ga-PSMA-HBED-CC) represents a successful novel PSMA inhibitor radiotracer which has recently demonstrated its suitability in individual first-in-man studies. The radiometal chelator HBED-CC used in this molecule represents a rather rarely used acyclic complexing agent with chemical characteristics favourably influencing the biological functionality of the PSMA inhibitor. The simple replacement of HBED-CC by the prominent radiometal chelator DOTA was shown to dramatically reduce the *in vivo* imaging quality of the respective ^68^Ga-labelled PSMA-targeted tracer proving that HBED-CC contributes intrinsically to the PSMA binding of the Glu-urea-Lys(Ahx) pharmacophore. Owing to the obvious growing clinical impact, this work aims to reflect the properties of HBED-CC as acyclic radiometal chelator and presents novel preclinical data and relevant aspects of the radiopharmaceutical production process of [^68^Ga]Ga-PSMA-HBED-CC.

## 1. Introduction

Early detection of metastases or recurrent prostate cancer (PC) lesions is of utmost clinical relevance in terms of clinical staging, prognosis and therapy management [1,2]. The high clinical impact of targeting the prostate-specific membrane antigen (PSMA) was recently demonstrated in a series of first-in-man examinations with either ^68^Ga- [3,4,5] or ^123^I-labelled [6] PSMA inhibitors. PSMA is a membrane-type zinc protease, also called glutamate carboxypeptidase II (GCPII), which is expressed by nearly all prostate cancers. Enhanced expression levels were found in poorly differentiated, metastatic and hormone-refractory carcinomas [7,8]. Since urea-based inhibitors of PSMA clear rapidly from the circulation and since only low levels of physiological PSMA expression were detected in a few organs like the brain, kidney, salivary gland and small intestine [7,9,10,11] PSMA represents an ideal biological target for high quality PET imaging of prostate cancer [12,13,14,15,16,17].

Initial clinical experiences with the ^68^Ga-labelled PET tracer Glu-NH-CO-NH-Lys-(Ahx)-[[^68^Ga]Ga(HBED-CC)] ([^68^Ga]Ga-PSMA-HBED-CC) suggest that this novel tracer detects PC relapses and metastases with higher contrast as compared to ^18^F-choline. In a retrospective study, the images of 37 patients who received both ^18^F-choline PET/CT and ^68^Ga-PSMA PET/CT were analyzed [3]. Especially at low PSA values, PET/CT images obtained with [^68^Ga]Ga-PSMA-HBED-CC showed more PC lesions as compared to ^18^F-choline. It was concluded that [^68^Ga]Ga-PSMA-HBED-CC represents an attractive new imaging agent for the detection of recurrent prostate cancer and metastatic spread.

The chelator HBED-CC (*N,N'*-bis-[2-hydroxy-5-(carboxyethyl)benzyl]ethylenediamine-*N,N'*-diacetic acid), represents a hitherto rarely used acyclic complexing agent especially allowing efficient radiolabelling with ^68^Ga even at ambient temperature [18,19]. By combining HBED-CC with the PSMA inhibitor Glu-urea-Lys, a favourable aromatic part is introduced into the radiotracer which was found to be a necessary requirement for a sustainable interaction with the PSMA receptor, putatively with the accessory hydrophobic pocket of the PSMA S1 binding site [15,20,21]. We have indeed shown in a preclinical study that the replacement of HBED-CC by DOTA (1,4,7,10-tetraazacyclododecane-*N,N′,N″,N′″-*tetraacetic acid) resulted in a molecule not able to image the tumour at all [20]. Moreover, besides these biological advantages, HBED-CC represents a highly effective chelator. Extraordinary high thermodynamic stability constants of >10^39^ were determined for the complexation of Ga with HBED [22]. As mentioned before the structure is acyclic and demands rather low energy for complex formation which allows fast labelling at ambient temperature [19,20]. Moreover, a high kinetic stability of the Ga-HBED-CC complex at physiological pH was reported [23] resulting in a stable complex *in vivo* [24] and in human serum for at least 72 h [25]. Thus, HBED-CC represents a highly attractive radiogallium chelator for high-stability labelling of radiopharmaceuticals. However, in contrast to other clinically well-established radiometal chelators, HBED-CC forms three NMR-distinguishable diastereomers (RR, RS and SS configurations at the amine nitrogens) during gallium complexation, whereas presumably the RR configuration is thermodynamically favoured [24]. Besides the influence of the temperature the formation of the diastereomers was reported to be pH- and concentration-dependent as well [23]. In a standard labelling protocol, [^68^Ga]Ga-PSMA-HBED-CC is incubated at a pH of ~4 and heated at 95 °C. The thermodynamically favoured diastereomer is formed; however, a small fraction of one of the other two diastereomers is still present in the labelling reaction and would be part of the final formulation prepared for the patient. Thus, it is of utmost importance to analyze the two major diastereomers according to their biological activity in cell-based assays. The aim of this study is to summarize hitherto existing radiochemical experiences with HBED-CC conjugated low-molecular weight compounds and to confirm that the configuration of Ga-HBED-CC does not influence the cell binding properties of the resulting PSMA-targeted radioligand [^68^Ga]Ga-PSMA-HBED-CC, especially since the chelator has high impact on the interaction with the aforementioned accessory hydrophobic pocket of PSMA. Owing to the high and growing clinical impact, this work aims to present novel important preclinical data of [^68^Ga]Ga-PSMA-HBED-CC and relevant aspects of its radiopharmaceutical production. The fully automated radiosynthesis as well as the essential quality control parameters according to current EU-GMP regulations for radiopharmaceuticals are given.

## 2. Experimental Section

### 2.1. Reagents and Chemical Syntheses

The chemicals were of analytical grade and were used without further purification. Analysis of the synthesised molecules was performed using reversed-phase high performance liquid chromatography (RP-HPLC; Chromolith RP-18e, 100 mm × 4.6 mm; Merck, Darmstadt, Germany) with a linear A–B gradient (0% B to 100% B in 6 min) at a flow rate of 4 mL/min (analysis) or 6 mL/min (purification). Solvent A consisted of 0.1% aqueous trifluoroacetic acid (TFA) and solvent B was 0.1% TFA in acetonitrile. The HPLC system (L6200 A; Merck-Hitachi, Darmstadt, Germany) was equipped with a UV and a gamma detector (Bioscan, Washington, DC, USA). UV absorbance was measured at 214 nm and 254 nm. Mass spectrometry was performed with a MALDI-MS Daltonics Microflex system (Bruker Daltonics, Bremen, Germany). The ^nat^Ga-labelled reference Glu-urea-Lys(Ahx)-[Ga(HBED-CC)] (DKFZ-GaPSMA-11) was purchased from ABX Advanced Biochemical Compounds (Radeberg, Germany) and dissolved in 40 μL CH_3_CN/H_2_O (1:1). 2.6 μg were characterised at RT by mass spectrometry using an Agilent 1200 HPLC-MS system connected to an Orbitrap Mass Spectrometer (Exactive, Thermo Fisher Scientific) on a Hypersil Gold C18 column (2.1 mm × 200 mm, 1.9 μm; Thermo Scientific, Bremen, Germany) eluted with a linear gradient (eluent A: 0.05% TFA in water; eluent B: 0.05% TFA in acetonitrile; 0%–30% B in 30 min at RT, flow rate: 0.2 mL/min, absorbance: λ = 214 nm). Full scan single mass spectra (positive mode) were obtained by scanning from *m/z* = 200–4000.

The peptide c(RGDyK) was synthesised as described previously [26]. HBED-CC was conjugated by reacting with 1.2 equivalents of HBED-CC-TFP-ester which was synthesised as previously described [19]. The reaction mixture was supplemented with two equivalents of DIPEA in *N*,*N*-dimethylformamide (DMF). After HPLC purification (vide supra) the product was treated with TFA at room temperature for one hour resulting in the final compound. NOTA (1,4,7-triazacyclononane-1,4,7-triacetic acid) conjugation was carried out by reacting the peptide with 3 equivalents of SCN-Bn-NOTA (purchased from Macrocyclics, Dallas, TX, USA) in 0.1 M sodium carbonate buffer (pH 9.5) for 20 h at room temperature. The reaction mixture was purified by semipreparative HPLC. Mass spectrometry was used to confirm the identity of the synthesised compounds (*m/z* (NOTA-c(RGDyK) = 1071.3 (calc. for [M+H]^+^ = 1071.2); *m*/*z* (HBED-CC-c(RGDyK) = 1035.2 (calc. for [M+H]^+^ = 1035.2)).

### 2.2. Preclinical Radiolabelling and Radiochemical Stability

#### 2.2.1. ^68^Ga-Radiolabelling

^68^Ga (half-life 68 min; β^+^ 89%; E_β+_ max. 1.9 MeV) was obtained from a ^68^Ge/^68^Ga-generator based on a pyrogallol resin support [27]. For radiolabelling, the conjugates (0.1–1 nmol in 0.1 M HEPES buffer, pH = 7.5, 100 μL) were added to a mixture of 10 μL HEPES (2.1 M in H_2_O) and 40 μL [^68^Ga]Ga^3+^ eluate. The pH of the labelling solution was adjusted using NaOH. The reaction mixture was incubated at room temperature or 95 °C, respectively. The radiochemical yield (RCY) was determined via analytical RP-HPLC and thin layer chromatography (ITLC, Pall, Crailsheim, Germany) with a mixture of 0.9% NaCl and methanol (5:1) as solvent. Free activity was complexed by supplementing with 10 μL 0.1 M EDTA. In case of non-conjugated chelators, 0.9% NaCl was used as solvent.

#### 2.2.2. *^nat^Ga-Complexes*

A 10 times molar excess of Ga(III)-nitrate (Sigma Aldrich, Munich, Germany) in 10 μL 0.1 N HCl was reacted with the conjugates (1 mM in 0.1 M HEPES buffer pH 7.5, 40 μL) in a mixture of 10 μL 2.1 M HEPES solution and 2 μL 1 N HCl for 2 min at room temperature or 95 °C, respectively.

#### 2.2.3. Radiochemical Stability

The radiochemical stability of the ^68^Ga-labelled compounds was determined by incubating in both phosphate buffered saline (PBS) and human serum at 37 °C. An equal volume of acetonitrile was added to the samples to precipitate serum proteins. Subsequently, the samples were centrifuged for 5 min at 13,000 rpm (Heraeus Picofuge fresco, Thermo Fisher Scientific Germany, Schwerte, Germany). An aliquot of the supernatant and the PBS sample was analysed by RP-HPLC. In addition, serum samples were run on a Superdex 75 5/150 GL gel filtration column (GE Healthcare, Munich, Germany) in order to analyse protein binding. To investigate the complex stability against human transferrin, a 400 μL aliquot of the ^68^Ga-labelled peptide was added to 250 μg apo-transferrin and incubated at 37 °C in PBS at pH 7. The complex stability was determined using a Superdex 75 GL 5/150 short column with PBS (pH 7) as eluent.

### 2.3. In Vitro Testing

#### 2.3.1. Cell Culture

LNCaP cells (metastatic lesion of human prostatic adenocarcinoma, ATCC CRL-1740) were cultured in RPMI medium supplemented with 10% fetal calf serum and Glutamax (PAA, Pasching, Austria). The cells were grown at 37 °C in an incubator with humidified air equilibrated with 5 % CO_2_. The cells were harvested using trypsin-ethylenediaminetetraacetic acid (trypsin-EDTA; 0.25% trypsin, 0.02% EDTA, all from PAA, Austria) and washed with PBS.

#### 2.3.2. Cell Binding and Internalisation

The competitive cell binding assay and internalisation experiments were performed as described previously [20]. Briefly, LNCaP cells (10^5^ per well) were incubated with the radioligand [^68^Ga]Ga-PSMA-HBED-CC in the presence of 12 different concentrations of analyte (0–5000 nM, 100 μL/well, ^nat^Ga-labelled Glu-urea-Lys(Ahx)-HBED-CC prepared either by reaction at room temperature (RT) or by reaction at 95 °C). After incubation, washing was performed using a multiscreen vacuum manifold from Millipore (Billerica, MA, USA). Cell-bound radioactivity was determined using a gamma counter (Packard Cobra II, GMI, Ramsey, MN, USA). The 50% inhibitory concentration (IC50) was calculated using a nonlinear regression algorithm (GraphPad Software, version 5.01, La Jolla, CA, USA). Experiments were performed in triplicate.

The specific cell uptake and internalisation was determined in another cell-based assay. Briefly, 10^5^ cells per well were seeded in poly-L-lysine coated 24-well cell culture plates 24 h before incubation. After washing, the cells were incubated with 25 nM of the radiolabelled compounds (either labelled at RT or 95 °C) for 45 min at 37 °C and at 4°C, respectively. Specific cellular uptake was determined by competitive blocking with the PSMA inhibitor 2-(phosphonomethyl)pentanedioic acid (500 μM final concentration, 2-PMPA, Axxora, Loerrach, Germany). Cellular uptake was terminated by washing 4 times with 1 mL of ice-cold PBS. Cells were subsequently incubated twice with 0.5 mL glycine-HCl in PBS (50 mM, pH = 2.8) for 5 min to remove the surface-bound fraction. The cells were washed with 1 mL of ice-cold PBS and lysed using 0.5 mL 0.3 N NaOH. The radioactivity of the probes was measured in a gamma counter. The cell uptake was calculated as per cent of the initially added radioactivity bound to 10^6^ cells [%IA/10^6^ cells].

### 2.4. Automated Synthesis

A fully automated synthesis module (Scintomics GRP, Fürstenfeldbruck, Germany) and its ControlCenter and GRP-Interface software were used to transfer the radiosynthesis of [^68^Ga]Ga-PSMA-HBED-CC into an environment suitable for clinical application. The ^68^Ge/^68^Ga-generator used for radiopharmaceutical production was purchased from IDB-Holland BV (Baarle-Nassau, The Netherlands). Disposable cassette kits and chemicals including the precursor PSMA-HBED-CC (DKFZ-PSMA-11) in GMP-compliant grade used for the radiosynthesis were obtained from ABX advanced biochemical compounds. A Dionex Ultimate 3000 HPLC system (Thermo Fisher Scientific, Dreieich, Germany) equipped with a Chromolith Performance RP-18e column (100 mm × 4.6 mm, Merck) and a NaI radiodetector (Raytest, Straubenhardt, Germany) was used to determine the radiochemical purity. The mobile phase consisted of gradient mixtures of acetonitrile (A) and 0.1% aqueous TFA (B); 0–0.5 min: 95% B; 0.5 to 10 min linear gradient to 80% A; flow rate 2 mL/min. Residual solvents were determined using a 6850 Series gas chromatograph (Agilent Technologies, Böblingen, Germany). Bacterial endotoxin testing was performed using the LAL test with an Endosafe^®^-PTS device (Charles River Laboratories, Wilmington, DE, USA). The radionuclide was identified by determination of the half-life (67.9 min) using a CRC-15R dose calibrator (Capintec, Ramsey, NJ, USA). Radionuclidic purity of the final product solution and separation cartridges was analysed using gamma spectrometry (HPGe Canberra GC 5020, Meriden, CT, USA). Sterility testing was realised at the Department for Infectiology of the Heidelberg University Hospital. 1.9 μg (2 nmol) of PSMA-HBED-CC were dissolved in a mixture of 1.5 M acetate buffer pH 4.5 (1 mL) and 1 M ascorbic acid (10 μL) and the mixture was transferred into the reaction vessel. The ^68^Ge/^68^Ga-generator was eluted with 10 mL of 0.6 M HCl and the eluate diluted with 9 mL of ultrapure water. The mixture was then transferred to a cation exchange cartridge (Macherey-Nagel PS-H+, Size M, Düren, Germany) and eluted with 5 M NaCl solution (1.2 mL) into the preheated reaction vessel (100 °C). The reaction mixture was heated for 10 min. The crude reaction mixture was then removed from the reaction vessel and transferred to a pre-conditioned (1. 10 mL EtOH/ 2. 10 mL ultrapure water) C18 cartridge (Waters Sep-Pak light, Eschborn, Germany). 9 mL ultrapure water was used to rinse the reaction vessel and passed over the C18 cartridge. The C18 cartridge was washed with another 5 mL of ultrapure water. The final product was eluted from the C18 cartridge with 2 mL of EtOH/H_2_O (1:1 v:v), sterile filtered (Millipore Cathivex-GV, 0.22 μm) and diluted with 8 mL of PBS solution (according to Ph. Eur. 8.0 (4005000)). All quality control tests except those for sterility and radionuclidic purity were determined prior release of the final product.

## 3. Results and Discussion

### 3.1. HBED-CC as Chelator for Highly Efficient Radiolabelling of Peptides

The acyclic radiometal chelator HBED-CC was reported to represent a highly effective ^68^Ga complexing agent mainly suitable for gentle room temperature-radiolabelling of antibodies and proteins [18,19,24]. However, less is known about labelling kinetics and stability issues of ^68^Ga-labelled HBED-CC-conjugated peptides. First insights in the quality of HBED-CC as labelling moiety of low-molecular weight compounds were given in the context of HBED-CC-conjugated PSMA inhibitors. We have recently reported that Glu-urea-Lys(Ahx)-HBED-CC can be labelled in less than 2 min with 99% radiochemical yield (RCY) at room temperature [20,28]. Here we summarise our PSMA-independent radiochemical experiences with the HBED-CC-conjugated peptide c(RGDyK) compared to the NOTA-labelled counterpart.

**Figure 1:**
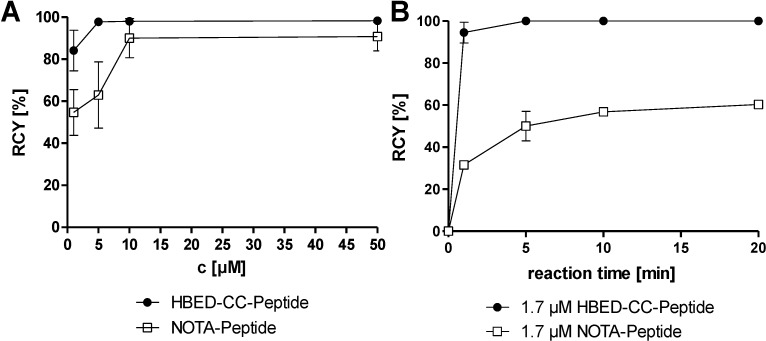
The radiochemical yields (RCY) of the HBED-CC- and NOTA-conjugated model-peptide c(RGDyK) as a function of (**A**) peptide concentration (2 min reaction time); and (**B**) reaction time (1.7 μM peptide concentration), respectively. The compounds were incubated with the generator eluate at room temperature in HEPES buffer (pH 4.2). The reaction was stopped by adding 10 μL of a 0.1 M EDTA solution and the reaction mixture was subsequently analysed via radio-HPLC (*n* = 6).

Figure 1A shows a typical dependency of the radiochemical yield (RCY) over time demonstrating the fast labelling kinetics of the examined HBED-CC-conjugated peptides at room temperature. A direct comparison of the labelling kinetics of a NOTA-conjugate and a HBED-CC-conjugate on the basis of proteins was already published previously [18] and is in good agreement with the herein presented data. In comparison to NOTA, HBED-CC complexes [^68^Ga]Ga^3+^ more efficiently at low concentrations and low temperatures. A concentration of 1.7 μM was found to be sufficient to form the ^68^Ga-labelled peptide with high radiochemical yields in less than one minute at room temperature. Clear differences were still observed after 20 min incubation of the reaction mixtures (Figure 1B).

The different labelling kinetics of HBED-CC and NOTA observed in these experiments were also confirmed in a *competition-for-chelation* assay performed with the HBED-CC- and NOTA-conjugated RGD-peptides. Equal concentrations of HBED-CC-c(RGDyK) and NOTA-c(RGDyK) were incubated with [^68^Ga]Ga^3+^ at room temperature. Also under these conditions, HBED-CC turned out to be the more potent chelator in direct challenging with NOTA, as 16% of the activity was complexed by NOTA-c(RGDyK) whereas 84% of the activity was incorporated in HBED-CC-c(RGDyK).

**Figure 2:**
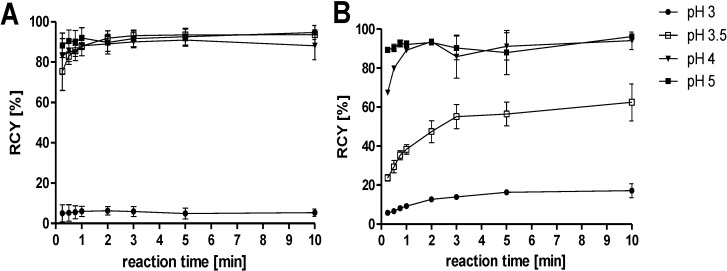
The pH dependence of the complexation reaction was determined by incubating the free acid of (**A**) HBED-CC (10 μM) and (**B**) NOTA (10 μM), respectively, at RT for 10 min. The radiochemical yield was determined by ITLC at the indicated time points (*n* = 6).

Labelling the free acid of HBED-CC was effective at pH values between 3.5 and 5 with no significant reduction of RCY between pH 4 and 5 (Figure 2). This fact ensures a high reproducibility and highly reliable radiopharmaceutical production process as a broad range of pH and an efficient radiolabelling at low concentrations of precursor enhance the robustness of the synthesis.

**Figure 3:**
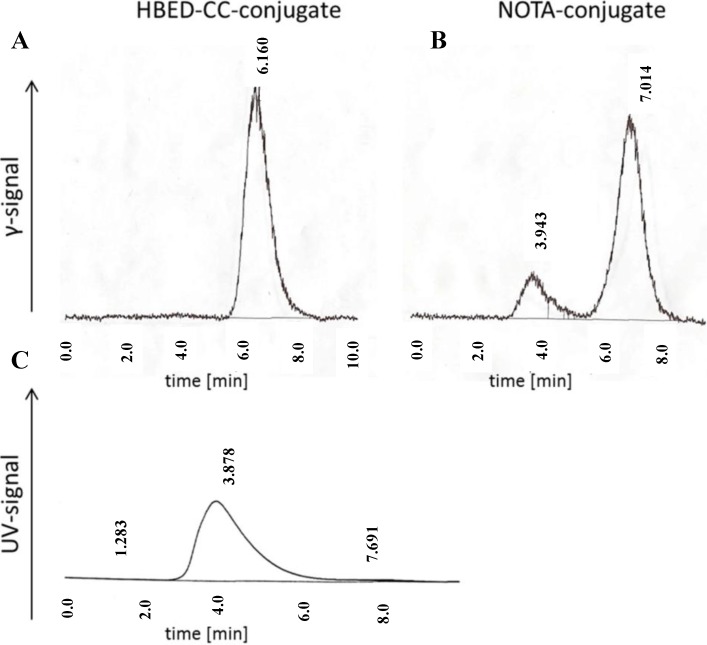
Superdex 75 5/150 GL runs of (**A**) ^67^Ga-labelled HBED-CC-c(RGDyK); and (**B**) ^67^Ga-labelled NOTA-c(RGDyK) after 48 h incubation in human serum at 37 °C. Only the radiometric signal is shown. The UV trace showed a major peak at ~3.9 min p.i.; (**C**) which corresponds with the elution time of serum proteins.

Another aspect which determines the quality and the clinical impact of a radiometal chelator is its serum stability. As metal complexes of open chain ligands generally show a lower degree of kinetic stability compared to cyclic chelators it is of importance to prove the long term stability of Ga-HBED-CC as active pharmaceutical ingredient of a radiopharmaceutical before its clinical use. The long term stability of radiogallium labelled HBED-CC-c(RGDyK) and NOTA-c(RGDyK) in human serum was investigated using the longer living radionuclide ^67^Ga. Neither the NOTA-conjugate nor the HBED-CC-conjugate showed non-complexed ^67^Ga in ionic form after 48 h-incubation in human serum at 37 °C. Remarkably, ~3% of the radioactivity was released from the NOTA-conjugate after 1 week in PBS at room temperature while the HBED-CC conjugate remained unchanged (>99% radiochemical purity (RCP)). This might be explained by a slight instability of the NOTA complex which was also demonstrated by analysing the serum samples on gel filtration (Figure 3). About 13% of the original peptide-bound radioactivity was eluted at about the same time as the serum proteins. In order to specify the transfer of ^67^Ga to the serum proteins, the stability was further investigated by incubating the ^68^Ga-labelled peptides in the presence of an excess of apo-transferrin for 2 h at 37 °C. However, no radiogallium was transchelated to apo-transferrin in both cases.

Taken together, HBED-CC represents an attractive acyclic alternative chelator for room temperature radiolabelling of proteins and peptides. Due to its different chemical characteristics compared to NOTA or DOTA such as the higher lipophilicity and the presence of aromatic residues, HBED-CC might have a positive impact on the pharmacokinetics or binding properties of a molecule. For [^68^Ga]Ga-PSMA-HBED-CC these chemical characteristics, obviously being a part of the pharmacophore, were shown to be advantageous for the PSMA binding behaviour of the molecule, presumably by interacting with the known accessory hydrophobic pocket of the PSMA S1 site [20].

### 3.2. The Influence of Diastereomers of HBED-CC on the Binding Properties

**Figure 4:**
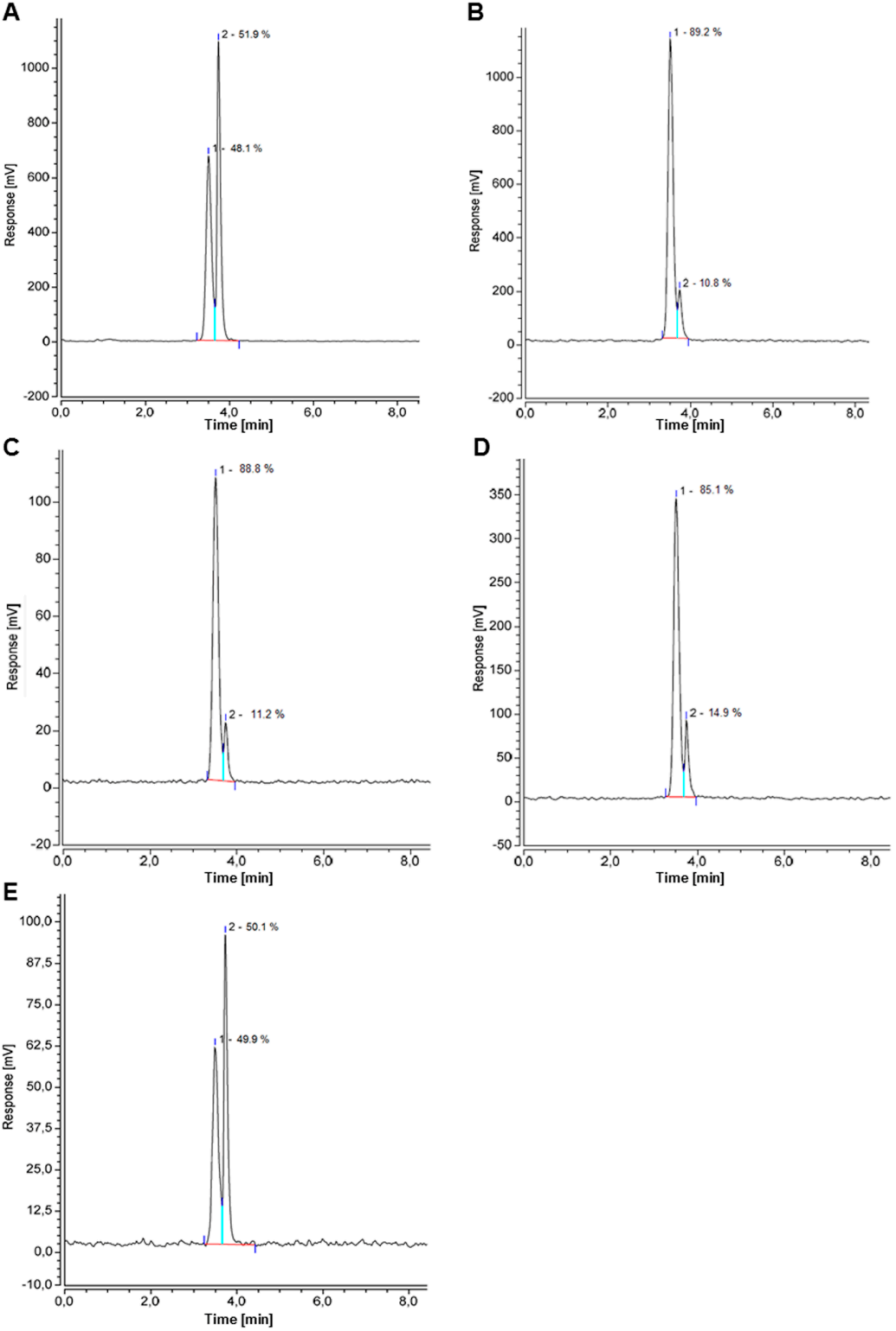
Radio-HPLC traces of RT (**A**) and 95 °C (**B**) labelled Glu-urea-Lys(Ahx)-HBED-CC. The peaks correspond to (1), the thermodynamically more stable, and (2), the thermodynamically less stable diastereomer, respectively. Graphs **C**–**E** show the radiochromatograms after various storage and labelling conditions: 95 °C labelled Glu-urea-Lys(Ahx)-HBED-CC stored for 3 h in injection buffer (**C**); RT labelled Glu-urea-Lys(Ahx)-HBED-CC after 3 h incubation in labelling reaction buffer at (**D**) pH 4, and (**E**) pH 7.

The active site of a PSMA inhibitor consists of two independent main binding sites, a zinc-containing rigid site and an efferent tunnel with rather lipophilic characteristics [29,30]. The classical binding motif of urea-based PSMA inhibitors typically interacts with its carboxylic groups and the carbonylic oxygen. For efficient internalisation of a PSMA-directed radiotracer, however, the interaction of the linker region of the molecule with the aforementioned rather hydrophobic tunnel region seems of crucial importance. HBED-CC turned out to presumably interact with this region as the replacement of DOTA by HBED-CC has shown a significant influence on the internalisation efficiency of [^68^Ga]Ga-PSMA-HBED-CC [20]. Due to this specific interaction with the hydrophobic binding pocket, slight chemical differences caused by the known formation of different diastereomers of HBED-CC after Ga-complexation might influence the binding properties of the whole molecule. The reaction temperature influences the proportional distribution of the three diastereomers. Typically, the GMP-compliant synthesis is realised at 100 °C resulting in the predominant formation of the thermodynamically more stable configuration of Ga-HBED-CC whereby the composition proved to be stable for at least 3 h in the injection buffer (Figure 4C). HPLC-MS measurements confirmed the identity of both diastereomers of the ^nat^Ga-labelled complex (supporting information). If the reaction is carried out at RT, a fraction of about 50% of another diastereomer is formed (Figure 4A). This diastereomer converts into the thermodynamically more stable one quite rapidly in a few hours at pH 4 (Figure 4D). However, neutralising the reaction mixture resulted in a much slower interconversion. As shown in Figure 4E the percentage composition remains nearly unchanged for hours which is in agreement with the observation of Schuhmacher *et al.*, who reported that the interconversion into the most stable configuration at pH 7 took several days [23].

**Figure 5:**
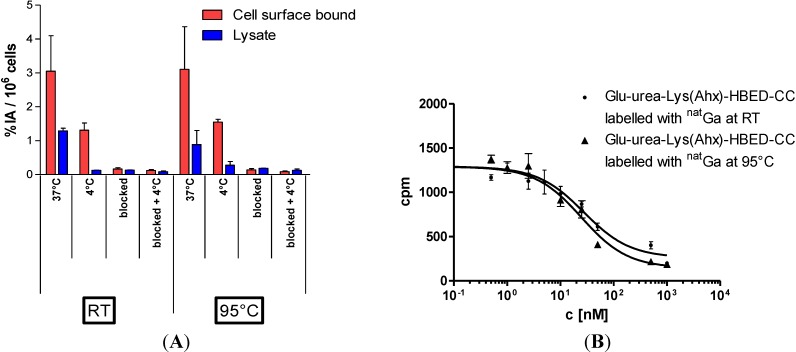
(**A**) LNCaP-cell binding and internalisation of [^68^Ga]Ga-PSMA-HBED-CC labelled at RT or 95 °C, respectively. Specific cell uptake was determined by competitive blockade with 500 μM of the PSMA inhibitor 2-PMPA. Values are expressed as percentage of applied radioactivity bound to 10^6^ cells [%IA/10^6^ cells]. Data are expressed as mean ± SD (*n* = 3); (**B**) Determination of binding affinity of [^nat^Ga]Ga-PSMA-HBED-CC to LNCaP cells as a function of the labelling temperature. The cells (10^5^ per well) were incubated with the radioligand (^68^Ga-labelled Glu-urea-Lys(Ahx)-HBED-CC) in the presence of different concentrations of ^nat^Ga-analyte (0–5000 nM, 100 μL/well).

From the *in vivo* point of view, the distribution of the diastereomers might be of high impact if one configuration of Ga-HBED-CC would not interact with the binding site and therefore would hamper the overall functionality of the PSMA inhibitor. Since the quality of the PET/CT images would be affected by the presence of a potentially non-functional diastereomer, it is important to elucidate their influence on the PSMA binding characteristics. Therefore, the cell binding properties of [^68^Ga]Ga-PSMA-HBED-CC labelled at ambient temperature and 95 °C, respectively, were evaluated in cell-based assays. Figure 5A shows that the PSMA-specific cell surface binding and internalisation of the mixture of diastereomers (RT labelling) and the thermodynamically most stable diastereomer (95 °C labelling) were comparable. In addition, the affinity related IC_50_-values of the reaction mixtures of both labelling conditions were determined on the PSMA expressing cell line LNCaP (Figure 5B). Indeed, both the RT-labelled fraction and the 95 °C-labelled fraction bound PSMA with identical affinities (IC_50_ values: 27.4 ± 1.3 nm and 24.8 ± 1.2 nm, respectively). It has to be concluded that the presence of a thermodynamically less stable diastereomer does not have any negative influence on the PSMA-binding properties indicating a robust stereochemical independence of the pharmacophore Glu-NH-CO-NH-Lys sidechain.

This information is important with regard to the radiopharmaceutical production process as a small fraction of one of the thermodynamically less stable diastereomers is still present in the labelling reaction mixture even at 95 °C labelling condition (Figure 4B) and would be part of the final formulation prepared for the patient. According to our results, a fraction of approx. 50% of the thermodynamically less stable diastereomer does not reduce the PSMA-specific cellular uptake and should therefore not have any negative influence on the quality of the PET images. However, it has to be considered that our conclusions are based on an indirect reasoning as the two major diastereomers were not separated chemically by HPLC for these experiments. A chemical separation via HPLC would result in an unknown concentration of unlabelled compound. As the cell binding directly depends on the used concentration it is crucial to know exactly the amount of analyte given to the cells. Since the proportional distribution of the observed two diastereomers can be easily controlled by pH and temperature of the labelling reaction mixture, the influence of the thermodynamically less stable diastereomer on the cell binding properties can be reliably determined by comparing RT-labelled and 95°C-labelled Glu-urea-Lys(Ahx)-HBED-CC. If the thermodynamically less stable diastereomer would not bind PSMA-presenting cell lines at all it would reduce the cell uptake considerably in the RT-labelled approach.

### 3.3. Automated Synthesis

The radiosynthesis of [^68^Ga]Ga-PSMA-HBED-CC as described earlier [20] was reproduced reliably and slightly modified on a fully automated synthesis module in 80% ± 5% decay corrected radiochemical yield within 35 min applying single-use cassette-based kits. An audit trail was recorded for each radiosynthesis including all performed steps and courses of radioactivity on three gamma detectors, gas flow and temperature of the heating unit. The steps involved include conditioning of the purification cartridge, elution of the ^68^Ge/^68^Ga-generator, purification of the generator eluate, radiosynthesis and purification and sterile filtration of the final product. [^68^Ga]Ga-PSMA-HBED-CC was obtained in >99% radiochemical purity as two diastereomers (Figure 4B) with a pH ranging between 6 and 8. Stability of [^68^Ga]Ga-PSMA-HBED-CC in the final product solution was tested for up to 2 h after the end of radiosynthesis and yielded identical results for the radiochemical purity. The only residual solvent found in the final product solution was ethanol in less than 10% v/v taking the density at 20°C to be 0.79 g/mL. Bacterial endotoxin testing showed <2 IU/mL and all samples tested were sterile. Filter integrity of the sterile filter was tested using the bubble-point test. Gamma spectrometry showed characteristic peaks at 0.511 and 1.077 MeV.

For the production of radiopharmaceuticals using gallium-68 obtained from a generator system it is crucial that possible metal impurities washed off from the ^68^Ge/^68^Ga-generator as well as ^68^Ge-breakthrough have to be eliminated prior the radiolabelling. Examples of methods for purification of the eluate are fractionated elution of the ^68^Ge/^68^Ga-generator or the trapping of the generator eluate on either a cationic or anionic exchange cartridge [31,32,33]. One of the most prominent methods has been described earlier by Zhernosekov *et al.* [33]. The drawback of this method for the purification of ^68^Ga is the use of acetone, which has to be removed after synthesis and bares the risk of additional impurities [34]. Recently, a method based on the use of a strong NaCl solution to purify ^68^Ga on a cation exchange cartridge has been reported [35,36]. For the radiosynthesis of [^68^Ga]Ga-PSMA-HBED-CC we adapted the method from Martin *et al.* [36]. A volume of 1.2 mL of 5 M NaCl solution was confirmed to elute up to 85% of the trapped ^68^Ga activity from the PS-H+ separation cartridge. As the majority of the ^68^Ga activity was eluted with the last 200 μL of NaCl solution, a smaller volume than 1.2 mL could not be applied. Larger volumes did not lead to higher elution efficiency. Remaining ^68^Ge was neither observed on the PS-H+ separation cartridge nor in the reaction vessel after elution with 5 M NaCl solution and neither in the final product preparation 48 h after purification of the generator eluate. This confirms the efficacy of this method to remove possible ^68^Ge-breakthrough. We modified the reaction parameters due to the larger geometry of the reaction vessel on the synthesis module compared to the method described by Eder *et al.* [20]. To allow good mixing of ^68^Ga and PSMA-HBED-CC in the reaction vessel, a minimum volume of 1 mL was necessary. Additionally, pH of the reaction mixture is seen to be a critical factor to obtain high radiochemical yields. HEPES (4-(2-hydroxyethyl)-1-piperazineethanesulfonic acid) is a well-known buffer in radiolabelling procedures using ^68^Ga, but as we consider HEPES as a critical impurity for radiopharmaceutical applications we were looking for alternative buffer solutions. Firstly, citrate buffer was applied at different concentrations and at different pH but this was unsuccessful resulting in radiochemical yields <5%. We used different sodium acetate buffer solutions (pH 3.2–5.5) in concentrations ranging from 20 mM to 1.5 M and volumes ranging from 1 to 3 mL. The optimal reaction conditions with an average radiochemical yield of 80% were found using 1 mL of 1.5 M sodium acetate buffer solution with a pH of the reaction mixture ranging from 3.6 to 4.2. Lower or higher pH of the reaction mixture lead to a significant decrease in radiochemical yields. Although the ^68^Ga-eluate was purified and concentrated using 5 M NaCl solution, sodium acetate buffer solutions with volumes smaller than 1 mL were not able to stabilise the pH of the reaction mixture sufficiently. Additionally, 10 μL of 1 M ascorbic acid was added to avoid the forming of side products when using a ^68^Ge/^68^Ga-generator with high starting activities [37]. We prolonged the reaction time to 10 min and heated to 100 °C because the resulting volume of the reaction mixture was by a factor 20 higher than previously reported [20]. High radiochemical yields were obtained reliably with only 1.9 μg (2 nmol) of PSMA-HBED-CC in our setup, different setups due to the properties of other synthesis modules might require slightly higher amounts (5–10 μg) of radiolabelling precursor. The crude reaction mixture was purified using a C18 cartridge which was then rinsed with ultrapure water to remove unreacted ^68^Ga. After sterile filtration 10 mL of the final product solution were obtained and 1.5–2 mL taken for quality control and keeping of a retain sample. Batches of [^68^Ga]Ga-PSMA-HBED-CC produced following this fully automated procedure are being released after passing quality control requirements according to a defined product specification (Table 1). The whole automated synthesis procedure was also successfully applied for the radiopharmaceutical production of [^68^Ga]Ga-DOTA-TOC and is a useful tool for a quick clinical implementation of other novel ^68^Ga-labelled compounds.

**Table 1:** Defined product specification of the final preparation of [^68^Ga]Ga-PSMA-HBED-CC.

Appearance	Clear and Colourless
pH	4–8
Radioactivity concentration	10–200 MBq/mL
Radiochemical purity (HPLC)	≥95%
Chemical impurities (HPLC)	≤5 μg/mL PSMA-HBED-CC
Concentration ethanol (GC)	<10% v/v
Approximate half-life	68 ± 6 min
Bacterial endotoxins	<17.5 IU/mL
Filter integrity (bubble-point test)	>3.5 bar
Radionuclidic purity (γ-spectrometry)	^68^Ga > 99.9% (γ-lines at 0.511 MeV and 1.077 MeV) ^68^Ge: ≤0.001%
Sterility	Sterile

## 4. Conclusions

We have recently introduced HBED-CC in the PSMA inhibitor motif Glu-urea-Lys as radiometal chelator in order to optimise the interactions of the pharmacophore with the accessory hydrophobic pocket of the PSMA S1 binding site. In addition, HBED-CC represents a very effective and stable radiometal chelator allowing fast radiolabelling at room temperature while exhibiting an exceptionally high complex stability similar to the clinically used DOTA chelator. The formation of diastereomers can be controlled via distinct temperature conditions during the radiolabelling reaction and directed predominantly to the formation of the thermodynamically more stable one. In case of small fractions of other diastereomers, the biological functionality of the radiotracer [^68^Ga]Ga-PSMA-HBED-CC is proven to be not affected. Taken together, the HBED-CC-conjugated PSMA inhibitor [^68^Ga]Ga-PSMA-HBED-CC is suitable for being used in typical kit like radiochemical production processes for clinical use with high reproducibility and robustness.

## Figures and Tables

**Figure S1:**
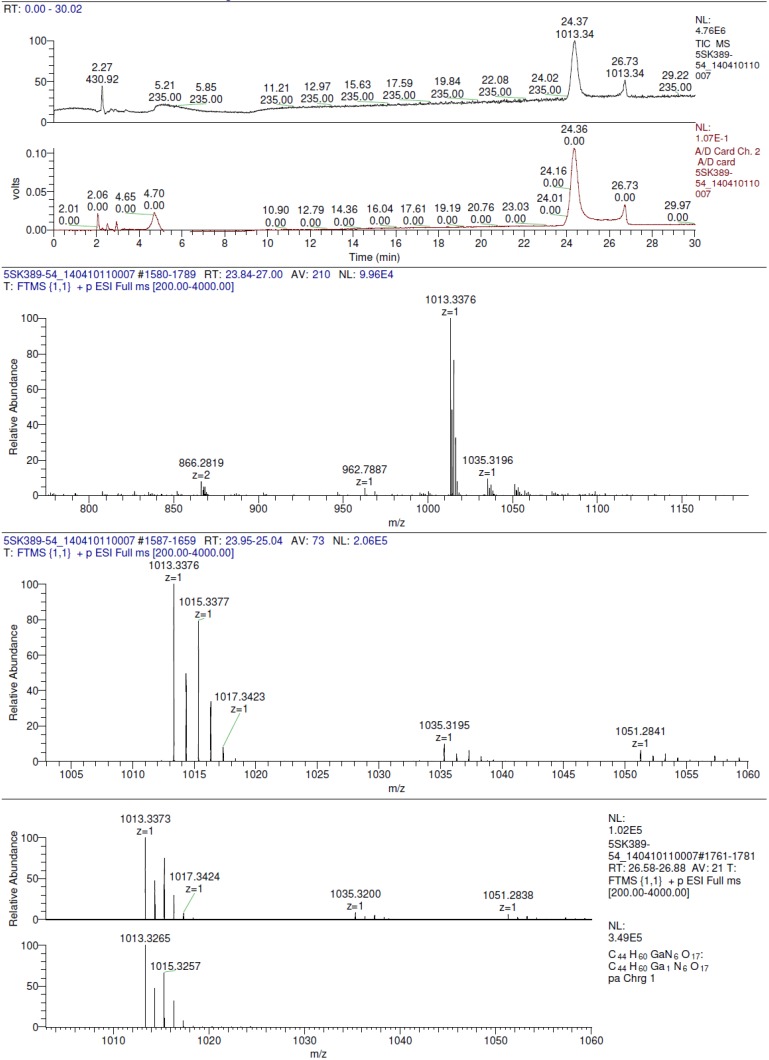
MS analysis of HPLC fractions of the mixture of diastereomers (fraction 23.84–27.00 min), of the thermodynamically more stable (fraction 23.95-25.04 min) and of the less stable diastereomer (fraction 26.58-26.88 min) of Ga-PSMA-HBED-CC, respectively.

## Appendix

### HPLC-MS analysis

The ^nat^Ga-labelled reference Glu-urea-Lys(Ahx)-[Ga(HBED-CC)] (DKFZ-GaPSMA-11) was purchased from ABX advanced biochemical compounds (Radeberg, Germany) and dissolved in 40 μL CH_3_CN/H_2_O (1:1). 2.6 μg were characterised at RT by mass spectrometry using a Agilent 1200 HPLC-MS system connected to an Orbitrap Mass Spectrometer (Exactive, Thermo Fisher Scientific) on a Hypersil Gold C18 column (2.1 × 200 mm, 1.9 μm; Thermo Scientific, Bremen, Germany) eluted with a linear gradient (eluent A: 0.05% TFA in water; eluent B: 0.05% TFA in acetonitrile; 0%–30% B in 30 min at RT, flow rate: 0.2 mL/min, absorbance: λ=214 nm). Full scan single mass spectra (positive mode) were obtained by scanning from *m/z* = 200−4000.
